# Supplementary material for: Light in, sound keys out: photoacoustic PUFs from stochastic nanocomposites
Source: Nat Commun. 2025 Aug 8;16:7323. doi: 10.1038/s41467-025-62747-1 (PMC12334560; doi:10.1038/s41467-025-62747-1)
Supplement: Supplementary file 1 — Supplementary Information [file 41467_2025_62747_MOESM1_ESM.pdf]

Supplementary information for

Light In, Sound Keys Out: Photoacoustic PUFs from Stochastic Nanocomposites

Taehyun Park [1,†], Junhyung Kim [2,†], Raksan Ko [1,†], Byullee Park [2, 3 ,4,*], and Hocheon Yoo [5,*]

[1]Department of Semiconductor Engineering, Gachon University, Seongnam 13120, Republic of Korea.

[2]Department of MetaBioHealth, Sungkyunkwan University, Suwon 16419, Republic of Korea

[3]Department of Biophysics, Institute of Quantum Biophysics, Sungkyunkwan University, Suwon 16419, Republic of Korea

[4]Department of Biopharmaceutical Convergence, Sungkyunkwan University, Suwon 16419, Republic of Korea

[5]Department of Electronic Engineering, Hanyang University, Seoul 04763, Republic of Korea

[†]T. Park, J. Kim, and R. Ko contributed equally to this work

[*]Correspondence should be addressed to: byullee@skku.edu (B.P), hocheon@hanyang.ac.kr (H.Y)

Table of Contents

Supplementary Note 3

Supplementary Note 1 Theoretical lateral resolution of the OR-PAM system. 3

Supplementary Figure 4

Supplementary Fig. 1 XPS spectra of CuO and SnO_2 nanoparticle films. 4

Supplementary Fig. 2 Surface SEM image and EDS analysis of the CuO film. 5

Supplementary Fig. 3 Surface SEM image and EDS analysis of the SnO_2 film. 6

Supplementary Fig. 4 Surface profile uniformity of the PA-PUF film. 7

Supplementary Fig. 5 Cross-sectional SEM analysis of PA-PUF film thickness. 8

Supplementary Fig. 6 Energy bandgap estimation of CuO and SnO_2 NPs using Tauc plots. 9

Supplementary Fig. 7 Schematic of spatial averaging in PA signal acquisition. 10

Supplementary Fig. 8 PA image obtained from CuO/SnO_2 mixed NP film. 11

Supplementary Fig. 9 PA images of individual CuO and SnO_2 NP films. 12

Supplementary Fig. 10 Entropy analysis of 10 different PA PUFs. 13

Supplementary Fig. 11 Characterization of the CuO NP film-based PUF device. 14

Supplementary Fig. 12 Characterization of the SnO_2 NP film-based PUF device. 15

Supplementary Fig. 13 Effect of CuO:SnO_2 ratio on PA-PUF randomness metrics. 16

Supplementary Fig. 14 Effect of NP concentration on PA-PUF randomness metrics. 17

Supplementary Fig. 15 Robustness of PA-PUF randomness under threshold variation. 18

Supplementary Fig. 16 Effect of binning size on PA-PUF randomness characteristics. 19

Supplementary Fig. 17 Randomness evaluation of the flexible PA PUF device. 20

Supplementary Fig. 18 Resistance of PA-PUF to machine learning-based modeling attacks. 21

Supplementary Fig. 19 PA signal stability under repeated laser exposure. 22

Supplementary Fig. 20 Effect of laser pulse frequency on PA-PUF randomness. 23

Supplementary Fig. 21 Mechanical robustness of the PA-PUF device. 24

Supplementary Fig. 22 Thermal stability evaluation of the PA-PUF response. 25

Supplementary Fig. 23 In-vivo movement stability of the PA-PUF response. 26

Supplementary Fig. 24 Noise reduction in PA imaging using bandpass filtering. 27

Supplementary Fig. 25 Stability of PA-PUF responses under ambient acoustic noise. 28

Supplementary Note

Supplementary Note 1 | Theoretical lateral resolution of the OR-PAM system.

In OR-PAM, lateral resolution depends on the optical components used in the system. A key parameter influencing resolution is the numerical aperture (NA) therefore, it is defined as follows.

$$NA = n \cdot sin(\theta) \tag{1}$$

ere n is the refractive index of the medium and θ is the half-angle of the maximum cone of light entering the system.

In this setup, the multimode fiber (MMF) has a core diameter of 10 μm and a numerical aperture of 0.10. The output beam from the fiber exhibits a divergence angle, given by the following expression.

$$\theta_{fiber\ output} = sin^{-1}(0.1) \approx 5.74^{\circ} \tag{2}$$

The collimator with a focal length of 11.75 mm transforms this diverging beam into a collimated beam with a diameter:

$$D_{collimated} = 2f_{collimator}tan(\theta_{fiber\ output}) \tag{3}$$

ing this collimated beam, the final numerical aperture after focusing with an objective lens of focal length 50 mm is substituting the values:

$$NA_{final} = \frac{D_{collimated}}{2f_{objective}} = \frac{f_{collimator}tan(\theta_{fiber\ output})}{f_{objective}} \approx 0.024 \tag{4}$$

Finally, the theoretical lateral resolution, determined by the Abbe diffraction limit for a wavelength of 532 nm, is expressed as follows.

$$Lateral\ Resolution = 0.51\frac{\lambda}{NA} = 11.3\ [\mu m] \tag{5}$$

Supplementary Figure

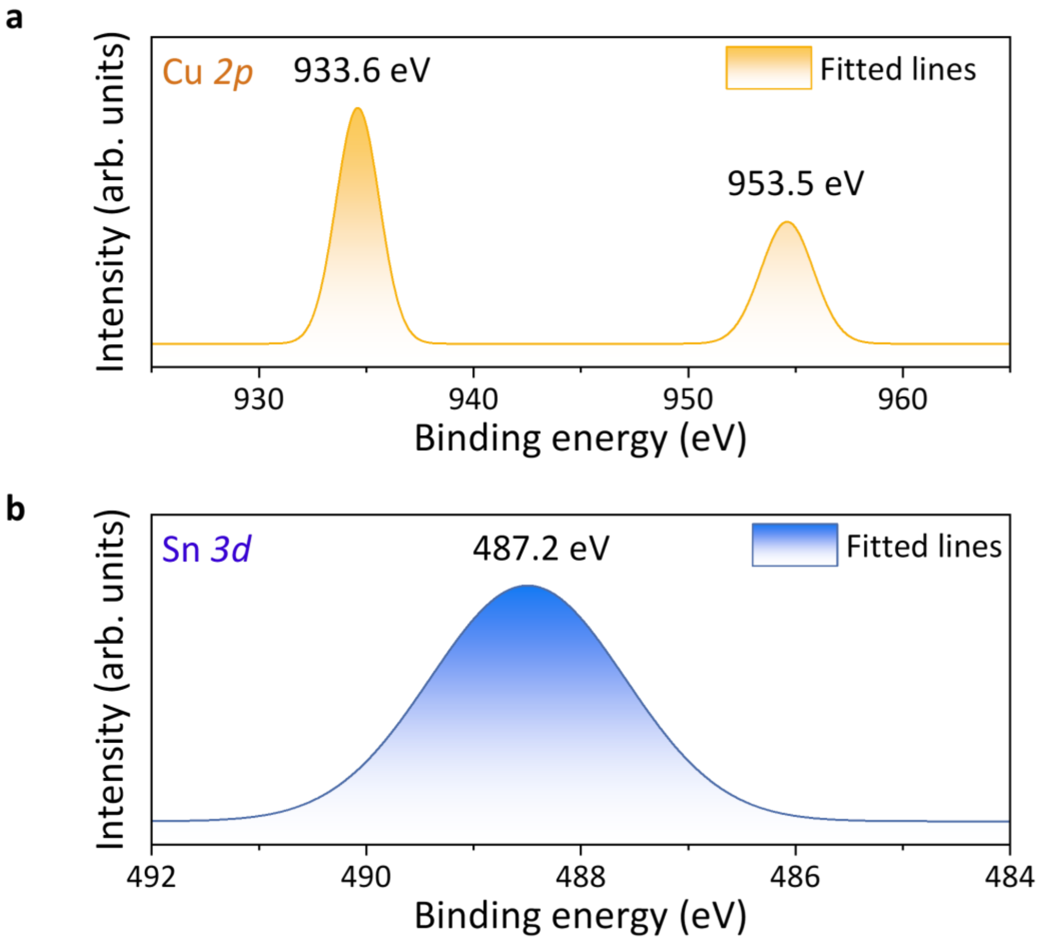

Supplementary Fig. 1 | High-resolution XPS spectra of CuO and SnO_2 nanoparticle films. XPS analysis focusing on the core-level regions of **a** Cu *2p* and **b** Sn *3d*, confirming the chemical composition of CuO and SnO_2, respectively.

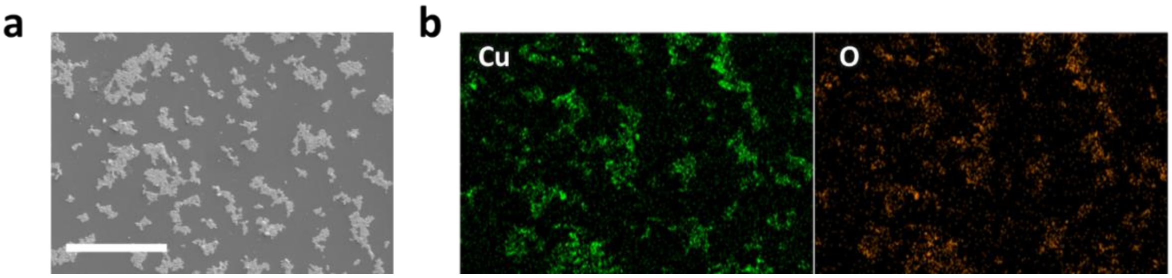

Supplementary Fig. 2 | Surface morphology and elemental composition of the CuO film. a SEM image of the CuO nanoparticle film surface. **b** Corresponding EDS elemental mapping indicating uniform Cu distribution. Scale bar: 100 μm.

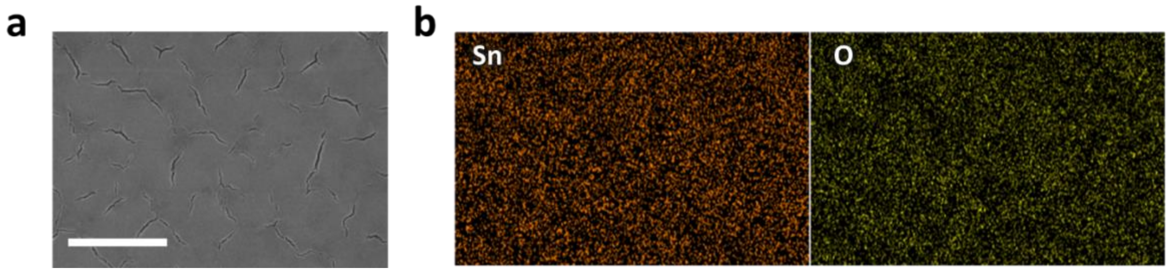

Supplementary Fig. 3 | Surface morphology and elemental composition of the SnO_2 film. **a** SEM image of the SnO_2 nanoparticle film. **b** EDS analysis showing Sn and O distribution across the film. Scale bar: 100 µm.

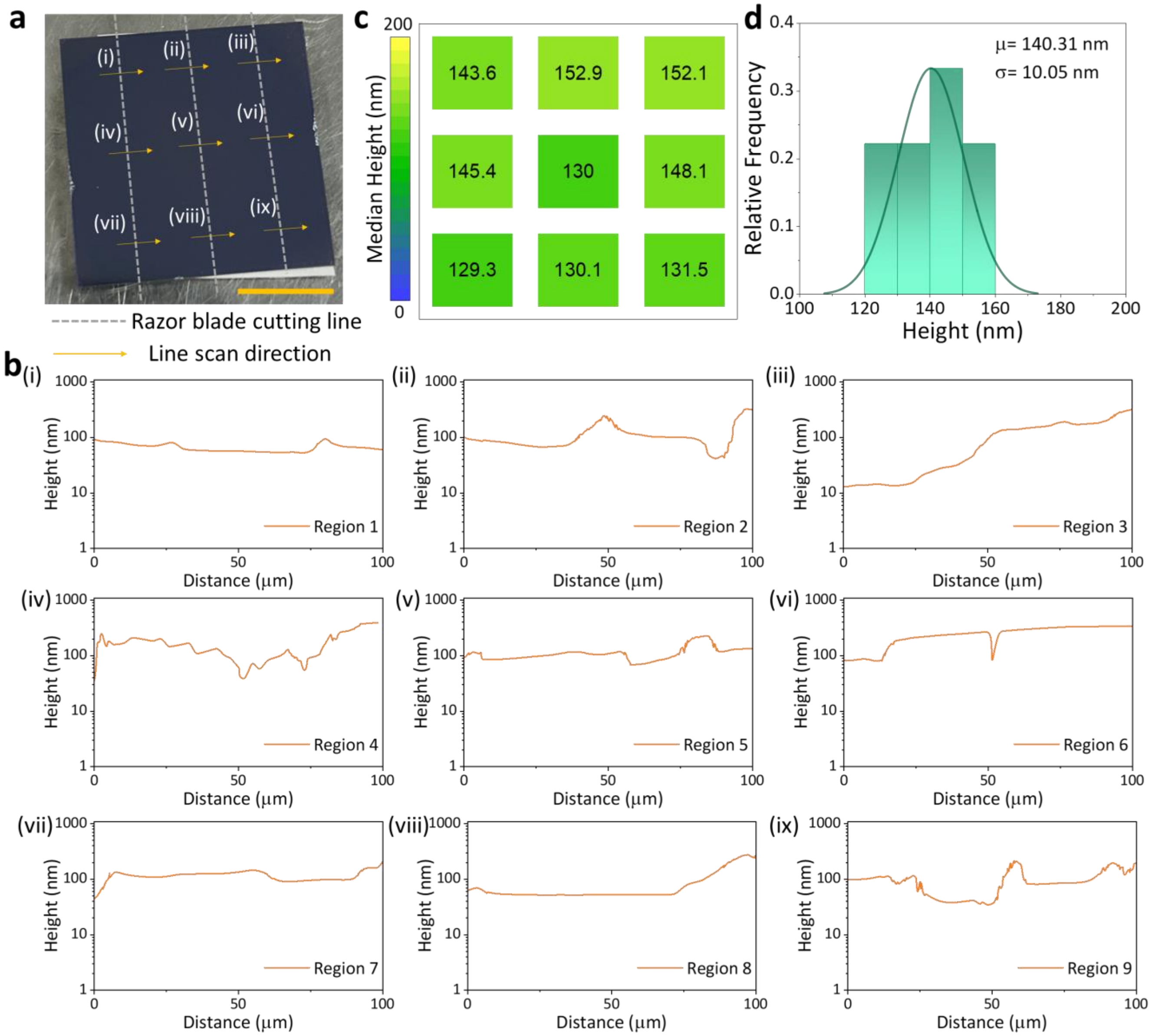

Supplementary Fig. 4 | Surface profile uniformity of the PA-PUF film. a Optical image of the film with nine marked regions for line scan. **b** Height profiles from each region (i–ix). **c** Median thickness values per region. **d** Overall thickness distribution (mean = 140.31 nm, SD = 10.05 nm).

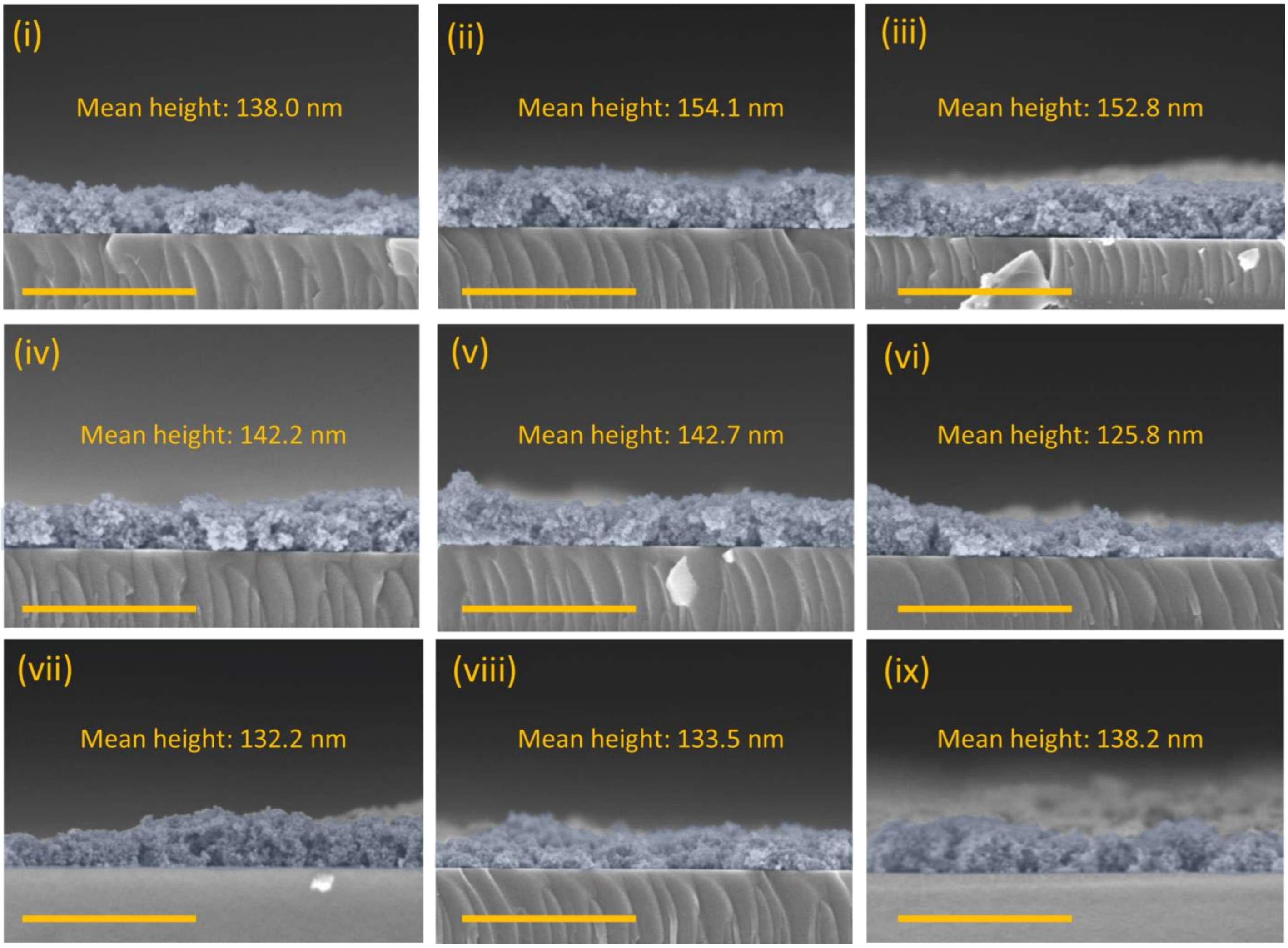

Supplementary Fig. 5 | Cross-sectional SEM analysis of film thickness uniformity. Cross-sectional SEM images showing thickness measurements from nine regions. Thickness values ranged from 125.8 nm to 154.1 nm. Scale bar: 500 nm.

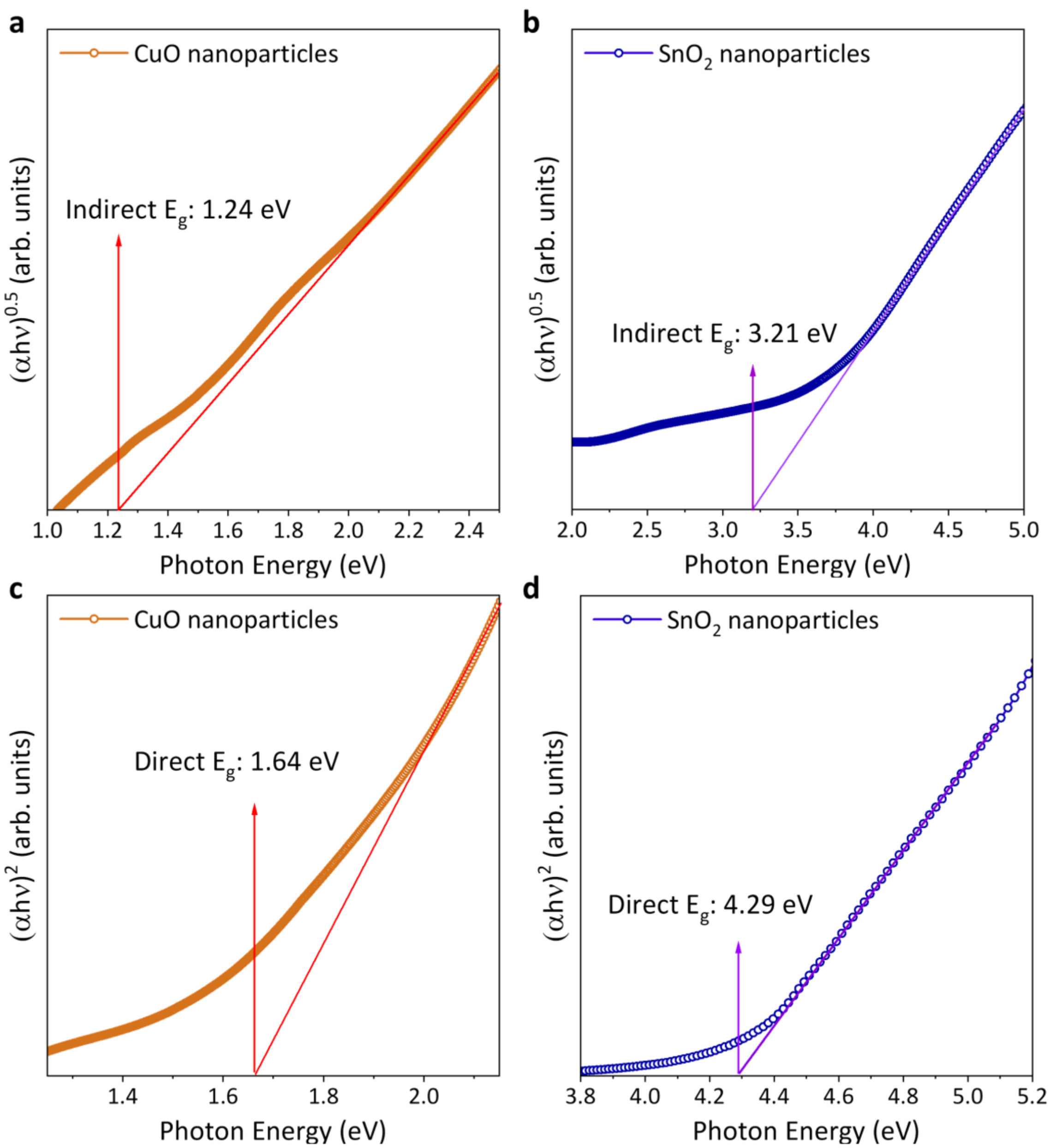

Supplementary Fig. 6 | Optical bandgap analysis of CuO and SnO_2 nanoparticles. **a** Tauc plot showing the indirect bandgap of CuO (1.24 eV). **b** Indirect bandgap of SnO_2 (3.21 eV). **c** Direct bandgap of CuO (1.64 eV). **d** Direct bandgap of SnO_2 (4.29 eV).

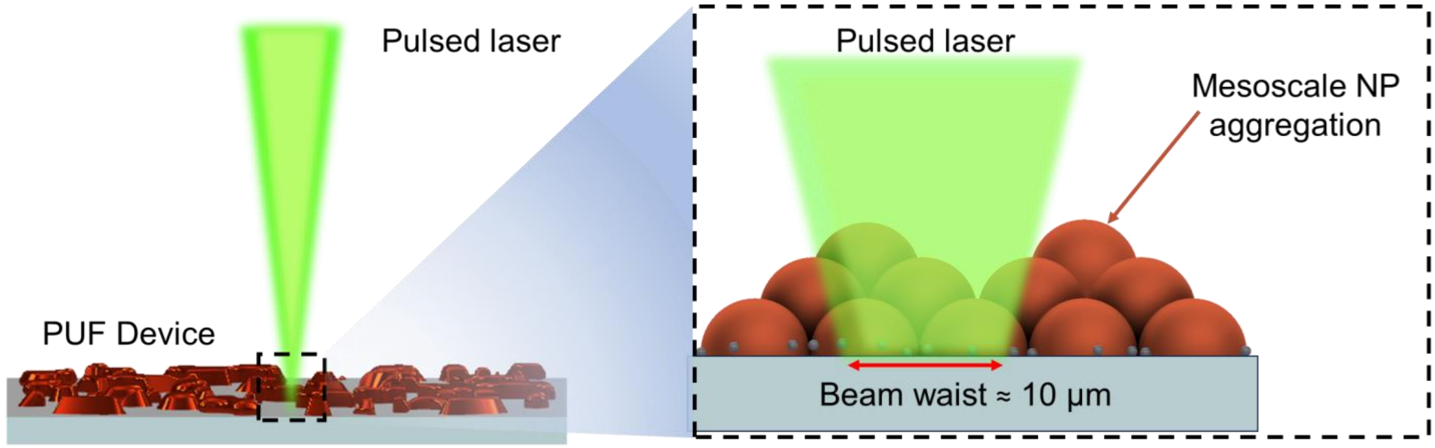

Supplementary Fig. 7 | Spatial resolution and sampling volume in PA excitation. Schematic illustration of the laser excitation geometry and spatial relationship between the beam size and nanoparticle distribution in the PUF device. The pulsed laser irradiates a heterogeneous surface composed of randomly aggregated CuO and SnO_2 nanoparticles. The beam waist (~10 μm) far exceeds the size of individual nanoparticles (<100 nm), resulting in spatial averaging within each pixel of the OR-PAM system.

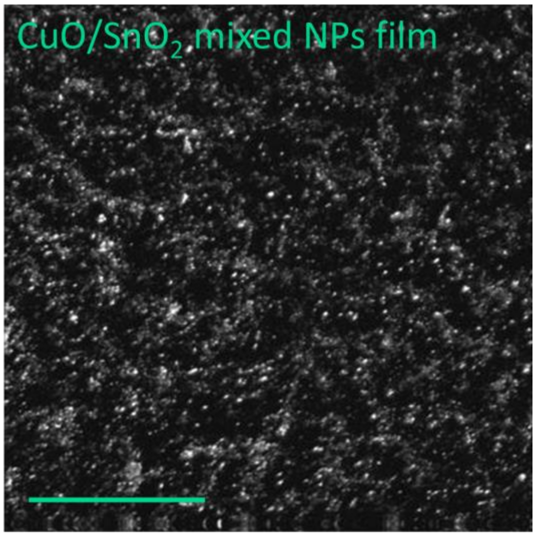

Supplementary Fig. 8 | Photoacoustic image of the mixed CuO/SnO_2 NP film. PA image acquired from the surface of the mixed NP film, demonstrating spatially resolved acoustic signal generation. Scale bar: 1 mm.

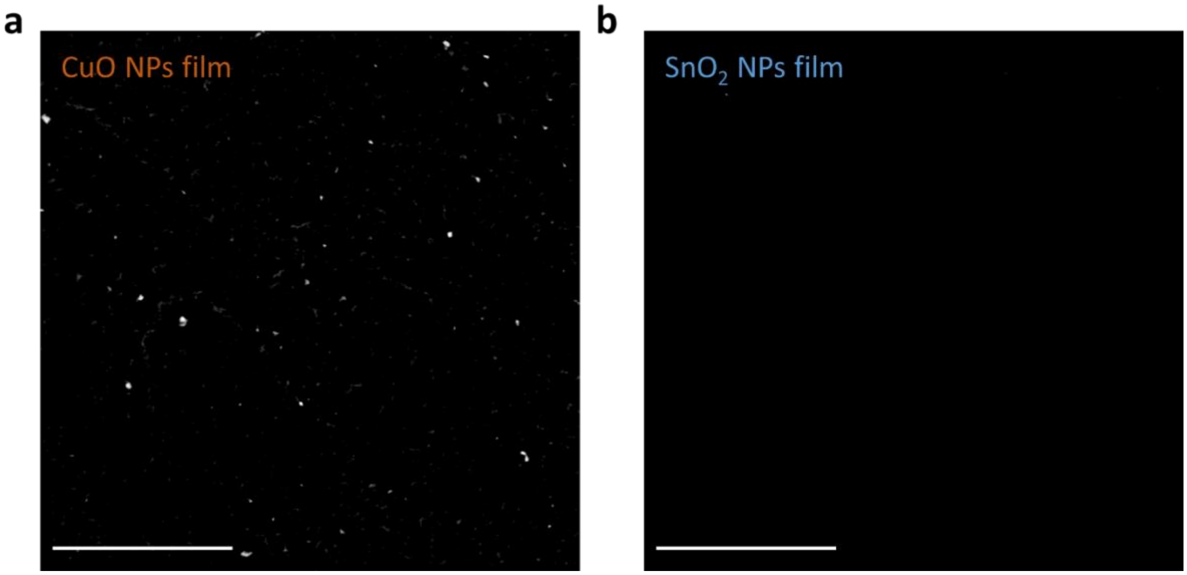

Supplementary Fig. 9 | Photoacoustic responses of individual NP films. PA images of **a** CuO and **b** SnO_2 films. Scale bar: 1 mm.

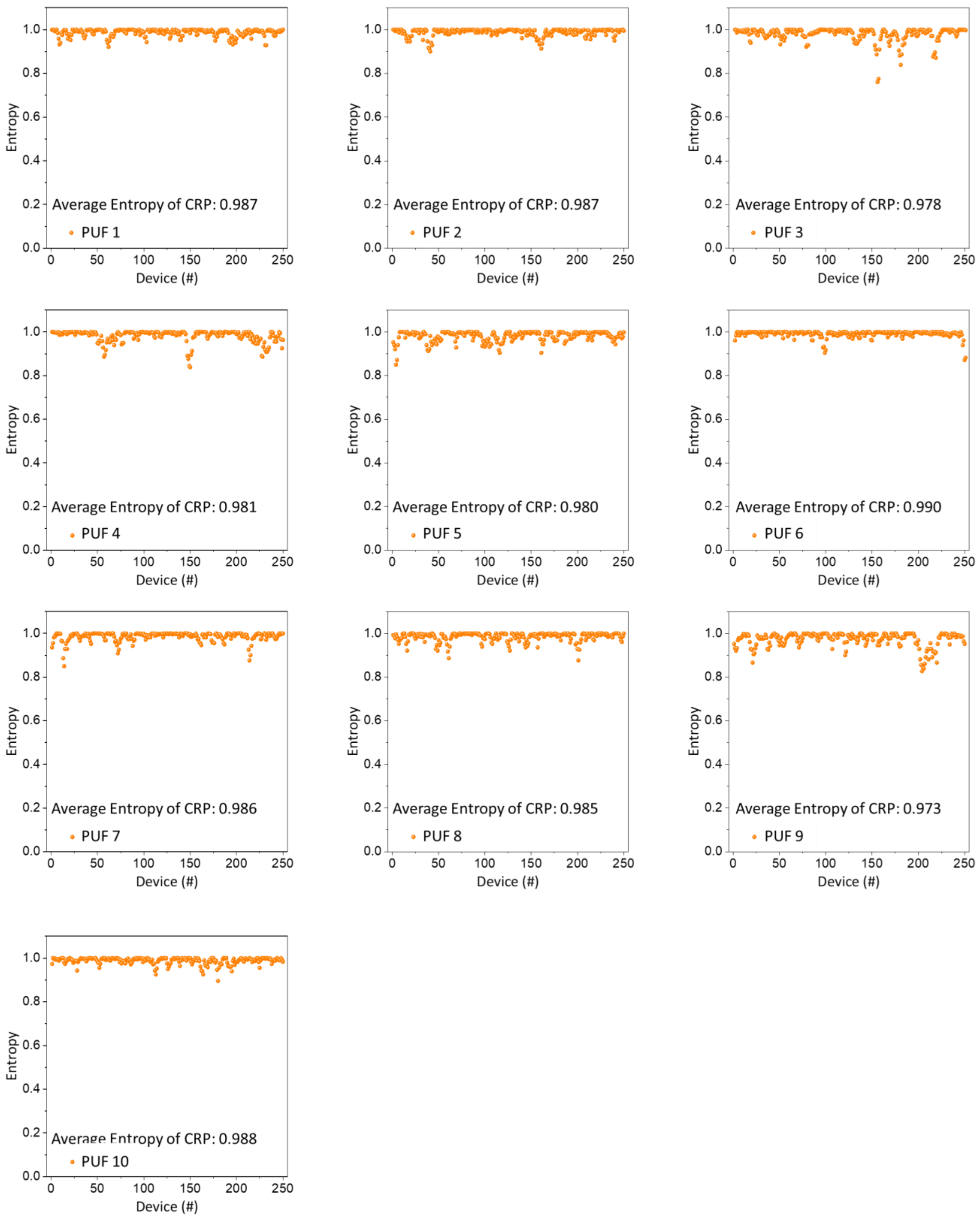

Supplementary Fig. 10 | Entropy analysis of PA PUF responses. Shannon entropy values calculated from 10 different PA PUFs.

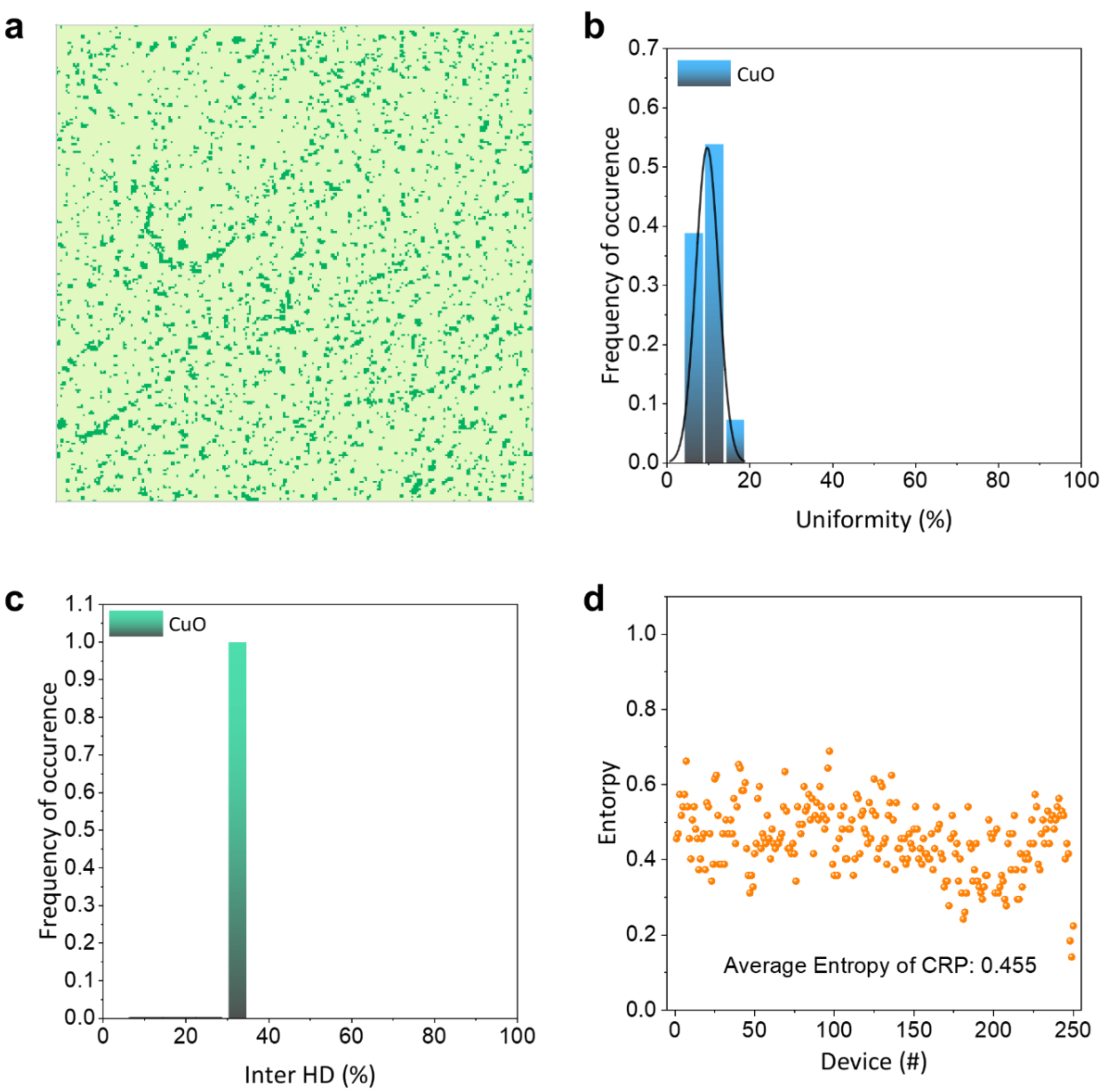

Supplementary Fig. 11 | Evaluation of the CuO NP-based PUF device. a Binary PUF pattern generated from the CuO film. **b** Uniformity. **c** Inter-Hamming distance. **d** Entropy analysis of the derived bitstream.

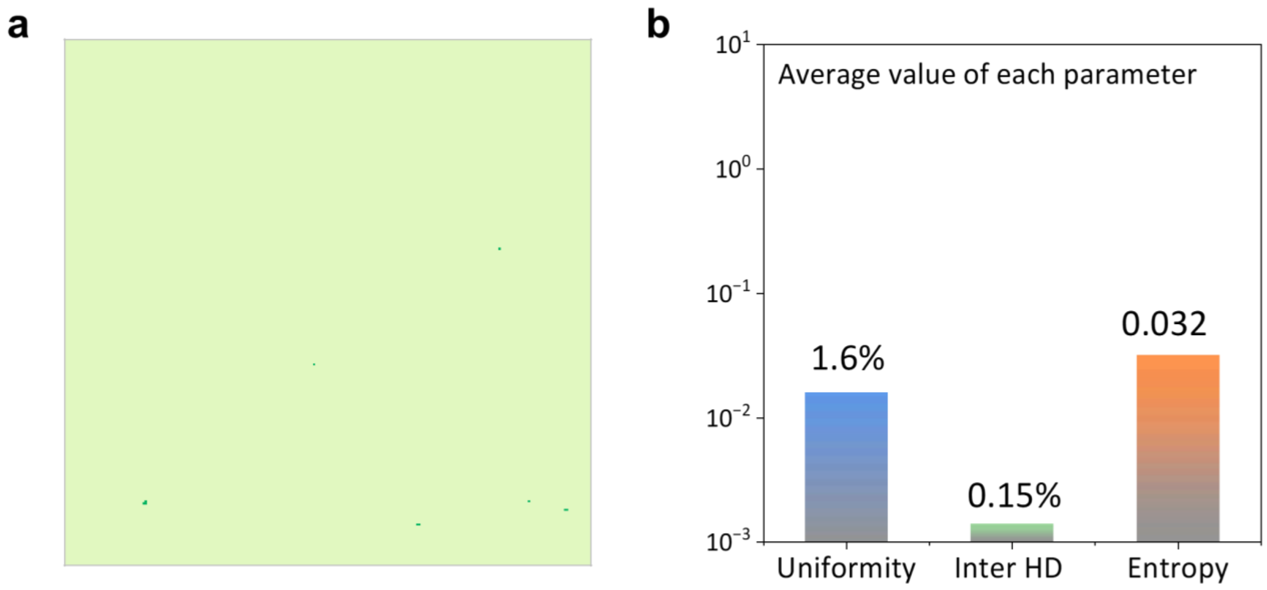

Supplementary Fig. 12 | Evaluation of the SnO_2 NP-based PUF device. a PUF response from the SnO_2 film. **b** Uniformity, inter-Hamming distance, and entropy evaluation results.

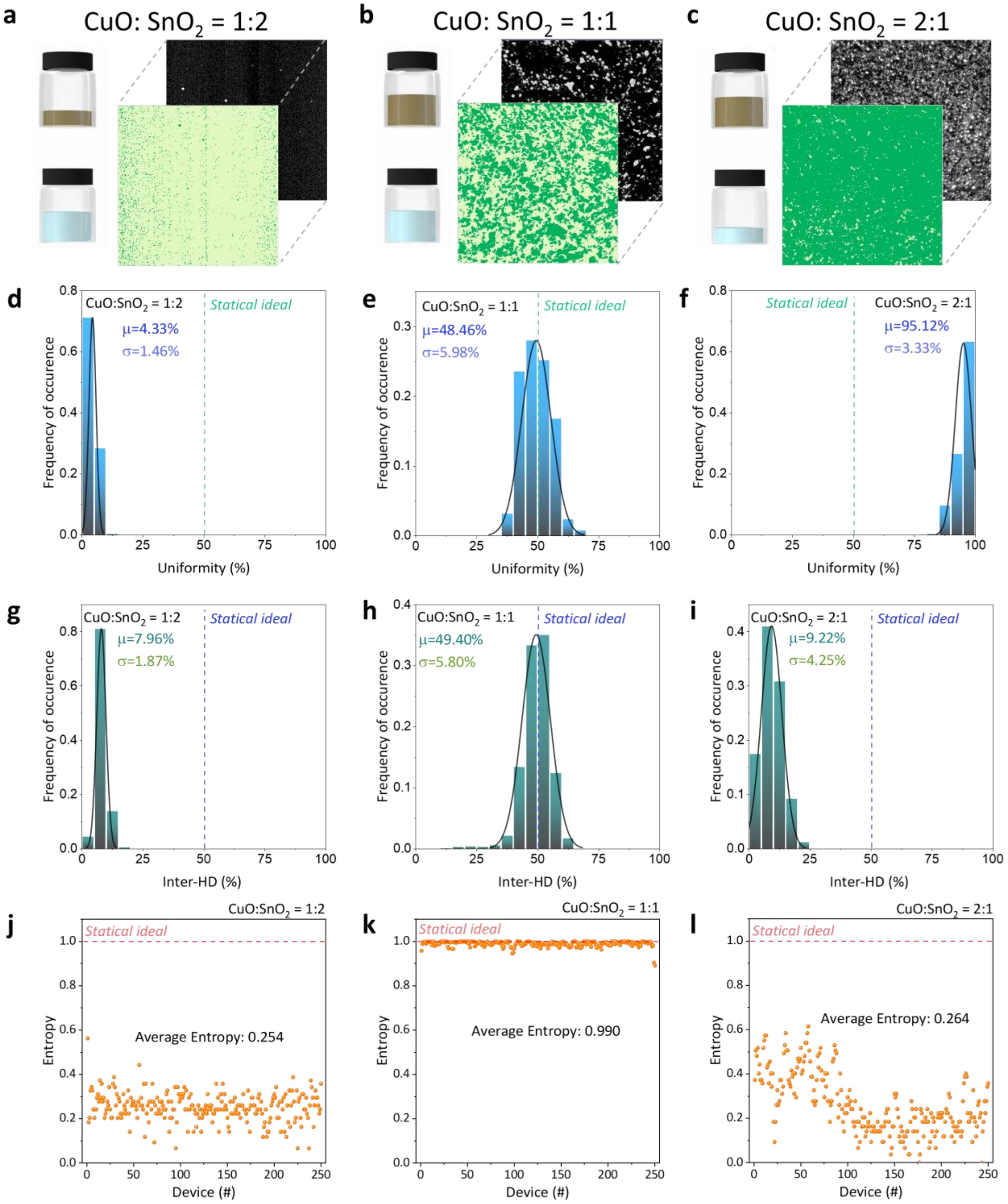

Supplementary Fig. 13 | Effect of $CuO:SnO_2$ mixing ratio on randomness characteristics. a–c PA-PUF response maps for mixing ratios of 1:2, 1:1, and 2:1. **d–f** Uniformity distributions for each composition. **g–i** Inter-HD distributions across devices. **j–l** Device-level entropy analysis.

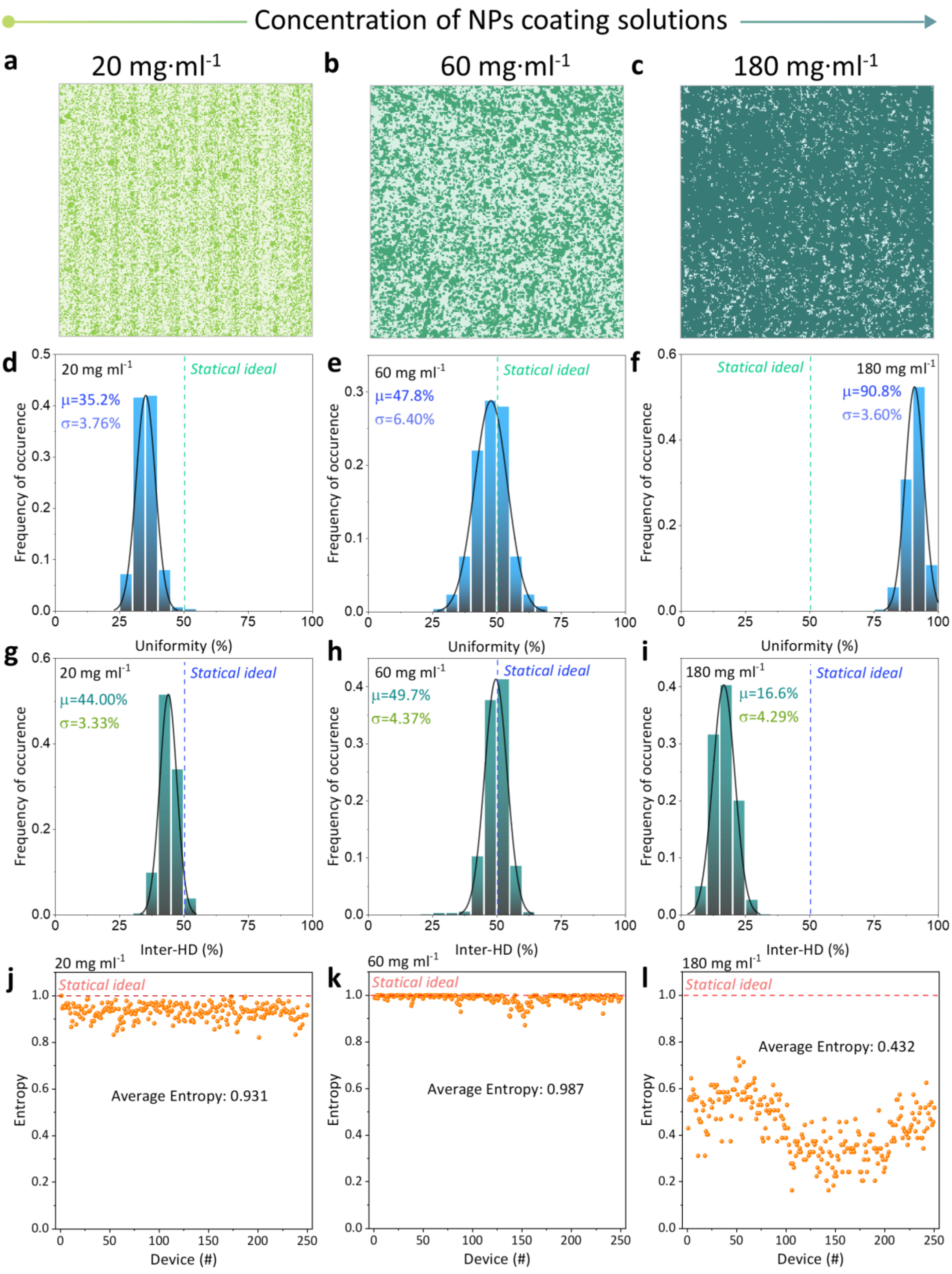

Supplementary Fig. 14 | Effect of total NP concentration on PA-PUF response randomness. a–c Binarized PA-PUF patterns for concentrations of 20, 60, and 180 mg·mL^{-1}. **d–f** Uniformity distributions. **g–i** Inter-HD distributions. **j–l** Entropy values across devices.

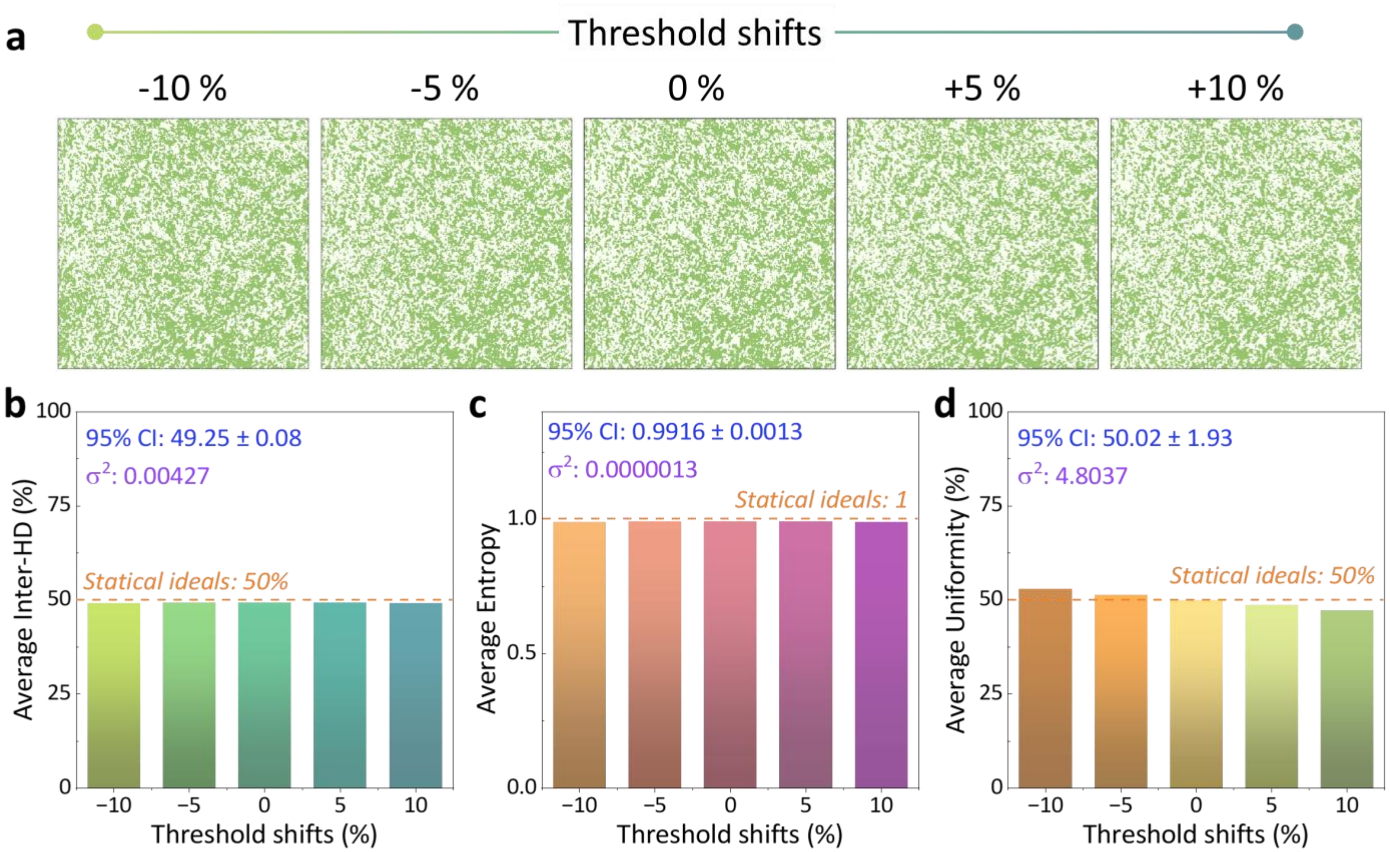

Supplementary Fig. 15 | Robustness of randomness against threshold variation. **a** Binary PA-PUF patterns generated with binarization thresholds of −10%, −5%, 0%, +5%, and +10%. **b** Inter-HD values under each threshold condition. **c** Entropy values showing consistency near the ideal value of 1. **d** Uniformity results demonstrating bit balance near 50% across all thresholds.

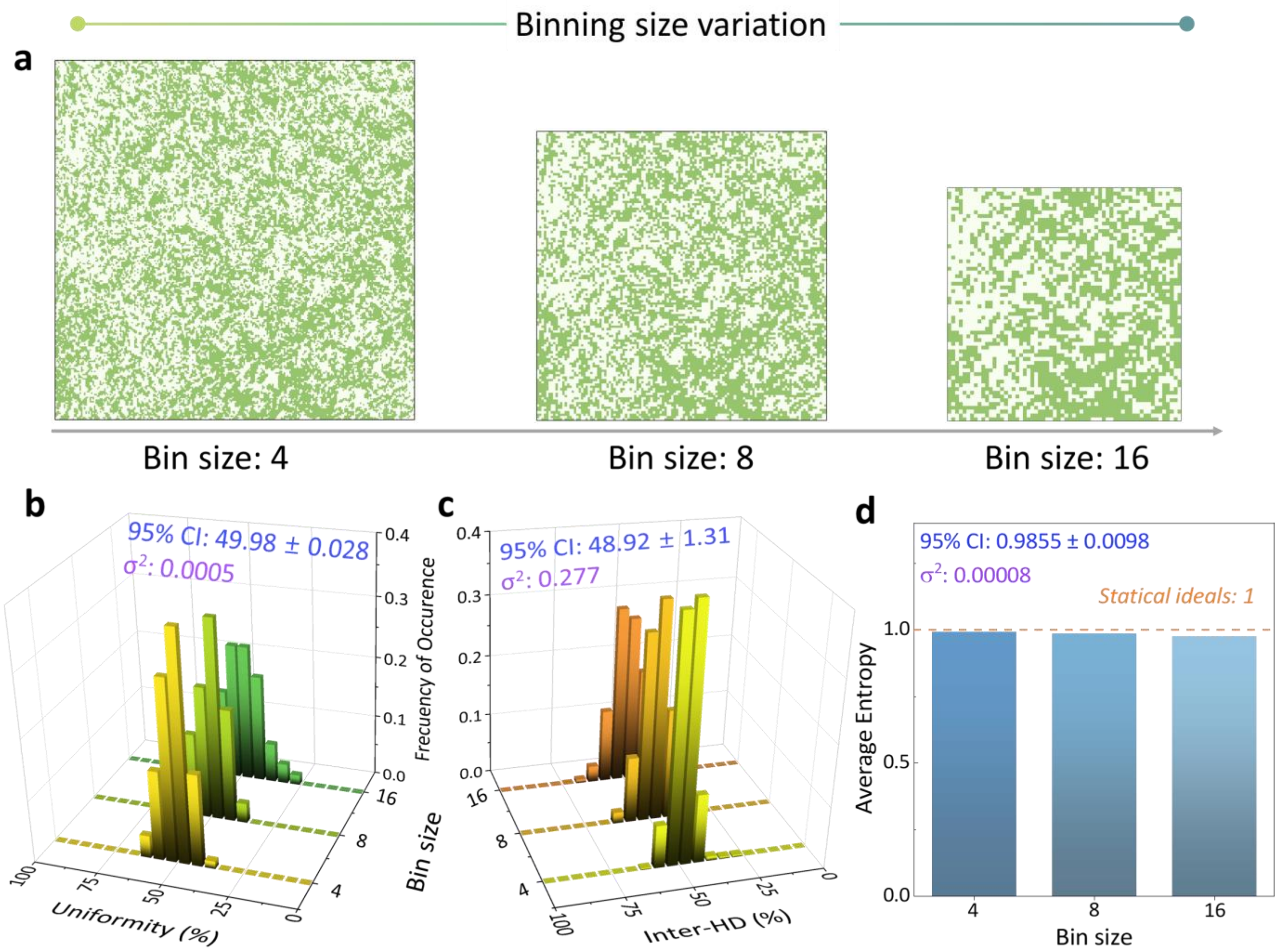

Supplementary Fig. 16 | Effect of binning size on PA-PUF randomness metrics. a Binary PA-PUF patterns generated using bin sizes of 4×4, 8×8, and 16×16. **b** Uniformity distributions with 95% CI of 49.98 ± 0.028%. **c** Inter-HD distributions with 95% CI of 48.92 ± 1.31%. **d** Entropy values with 95% CI of 0.9855 ± 0.0098.

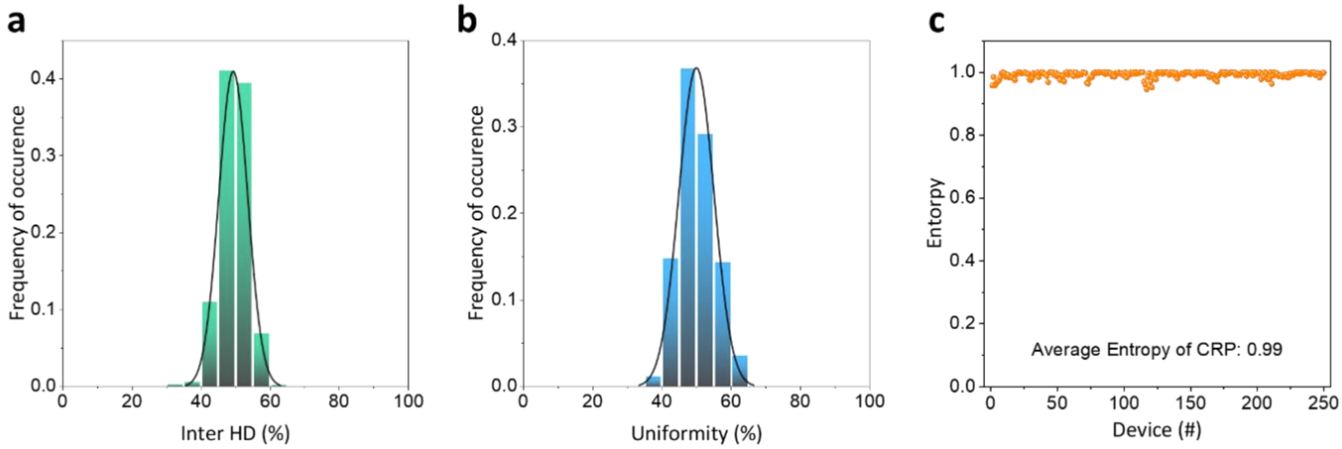

Supplementary Fig. 17 | Randomness evaluation of the flexible PA PUF device. a Inter-Hamming distance. **b** Uniformity. **c** Entropy results confirming statistical performance of the conformal, flexible PA PUF.

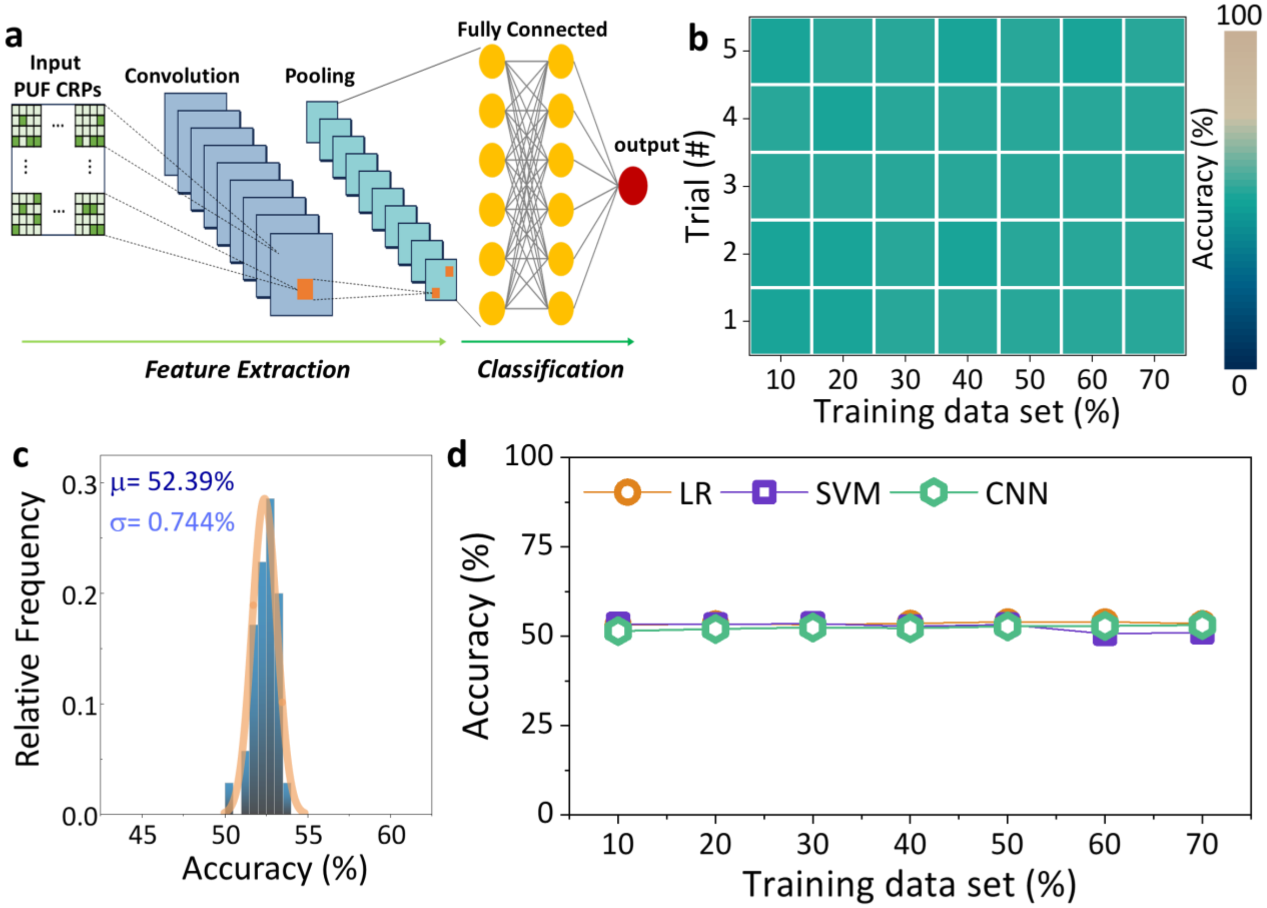

Supplementary Fig. 18 | Resistance of PA-PUF to machine learning-based modeling attacks. a Schematic of CNN-based attack architecture. **b** Heatmap of CNN prediction accuracy across training ratios from 10% to 70%. **c** Accuracy distribution across repeated trials. **d** Comparison of LR, SVM, and CNN prediction accuracy under matched conditions.

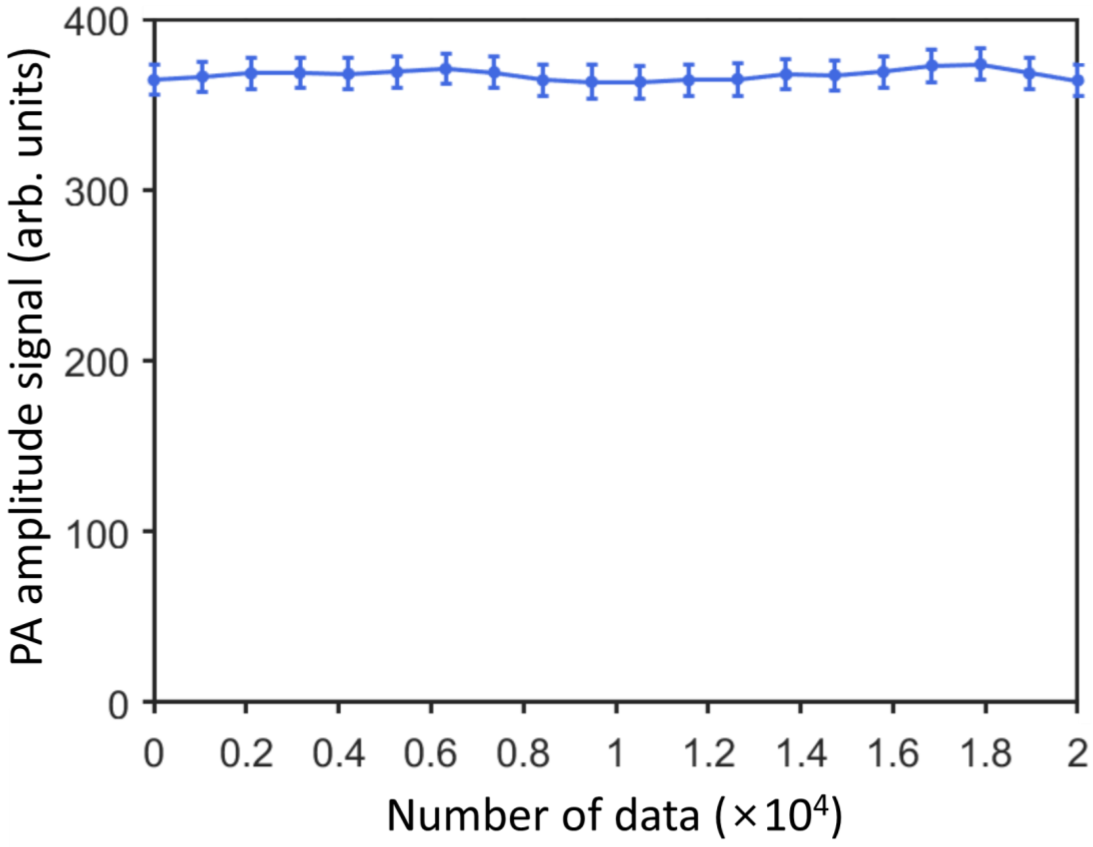

Supplementary Fig. 19 | Stability of PA signal amplitude under repeated laser exposure. PA signal amplitude measured at a fixed location over 20,000 consecutive pulses.

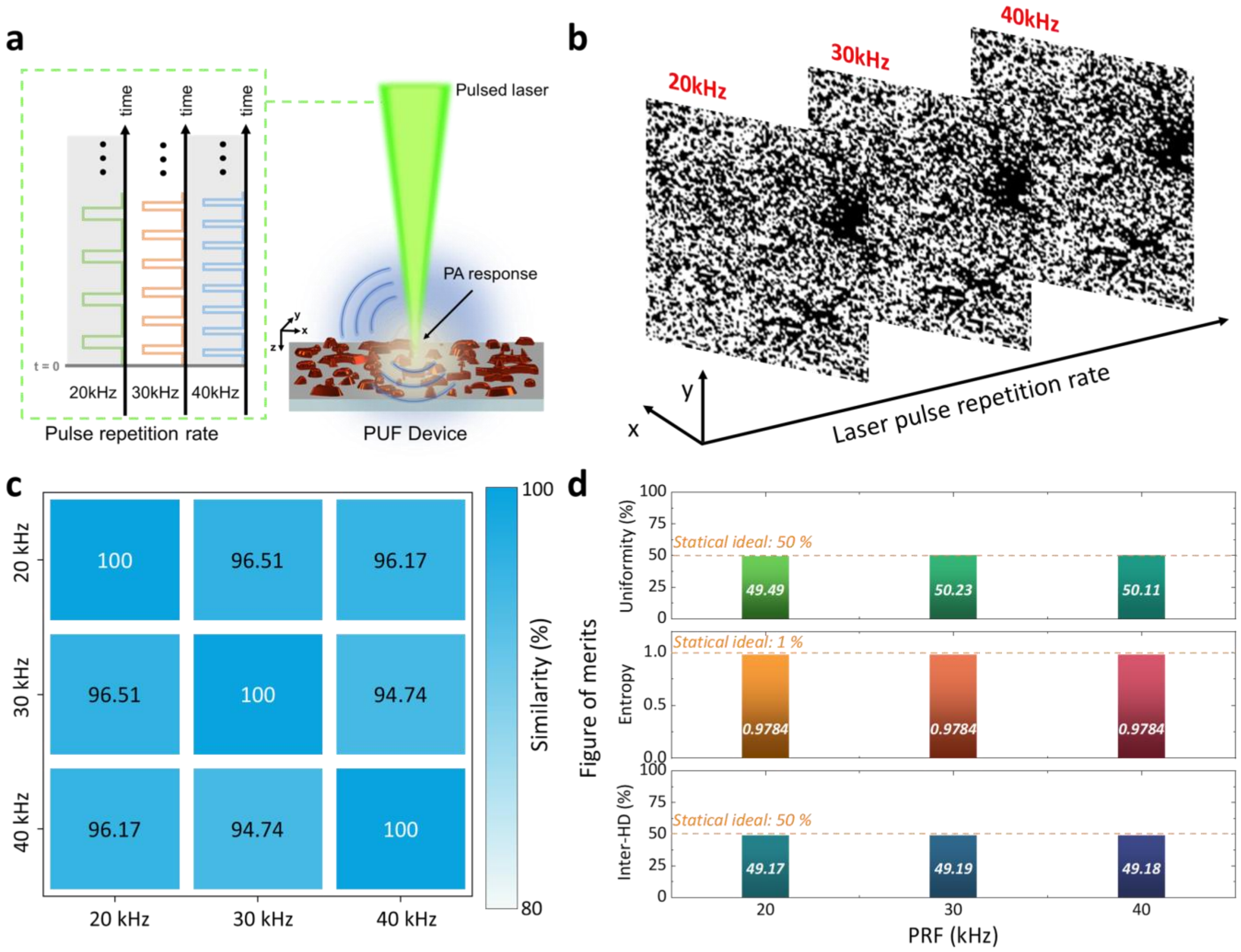

Supplementary Fig. 20 | Effect of laser frequency on randomness characteristics. **a** Schematic illustration of the experimental setup. **b** Digitized PA-PUF patterns generated under 20, 30, and 40 kHz PRF conditions. **c** Similarity analysis of repeated responses. **d** Statistical metrics including uniformity, entropy, and inter-HD.

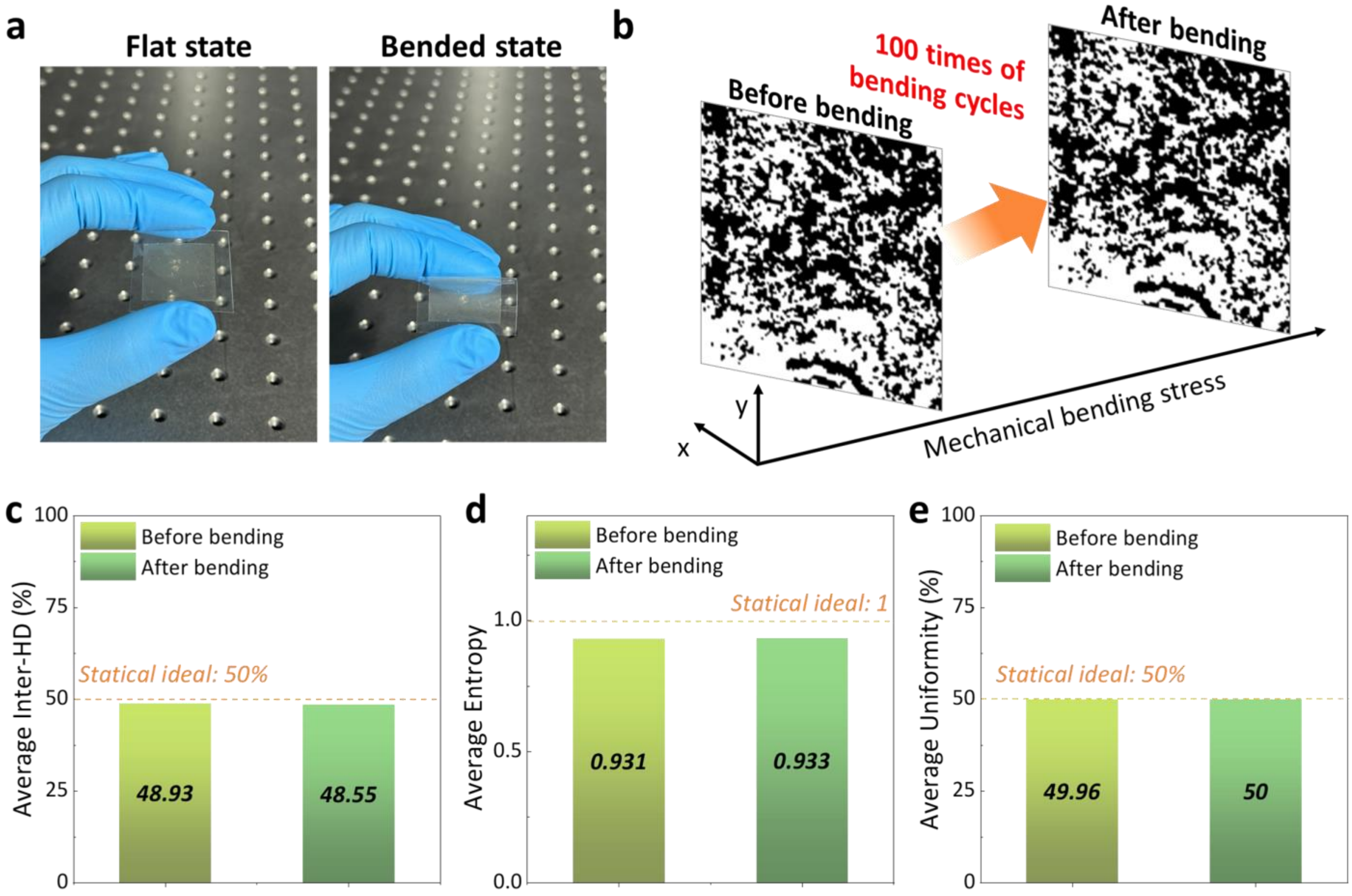

Supplementary Fig. 21 | Mechanical robustness of the PA-PUF device. a Photographs of the mechanical deformation test setup. **b** Representative digitized PA-PUF patterns. Statical analysis results of **c** inter-HD, **d** entropy, and **e** uniformity.

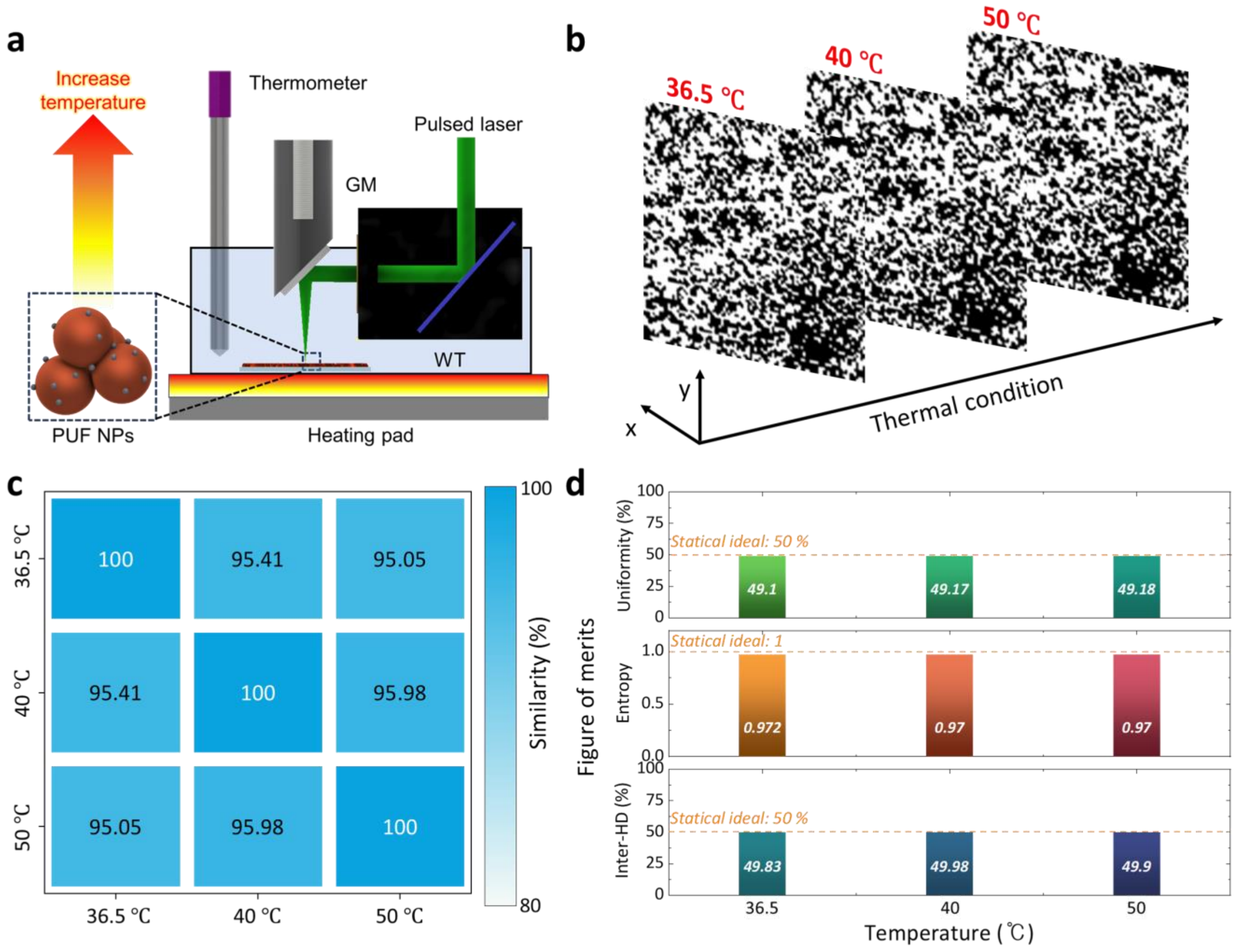

Supplementary Fig. 22 | Thermal stability evaluation of the PA-PUF response. **a** Schematic of thermal test setup. **b** Representative digitized PA-PUF patterns across temperature conditions. **c** Similarity analysis of thermal condition-based PUF responses. **d** Statical analysis results of uniformity, entropy, and inter-HD.

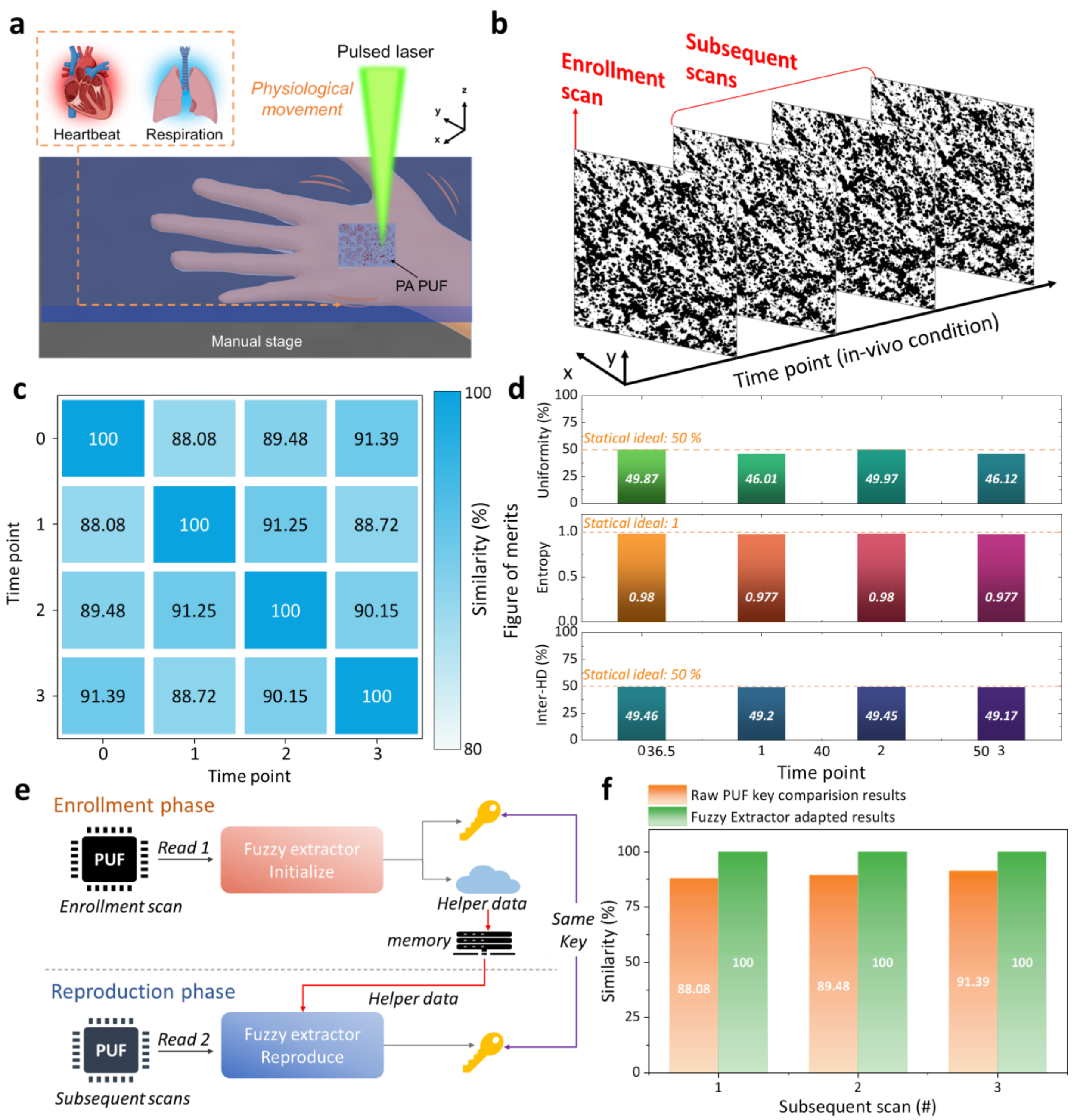

Supplementary Fig. 23 | Evaluation of PUF key stability under in-vivo motion conditions and recovery using a fuzzy extractor. a Schematic representation of repeated PA pattern acquisition with the PUF device attached to human skin under in-vivo motion conditions. **b** Obtained PA PUF patterns. **c** Similarity matrix showing pairwise bit-level comparisons among the four scans. An average bit error rate of 10.35% was observed between the enrollment and subsequent scans. **d** Uniformity distribution, entropy analysis, and inter-HD distribution of each PA PUF responses. **e** Conceptual diagram of fuzzy extractor implementation. During enrollment, a key and helper data are generated; in the reproduction phase, noisy inputs are corrected using the stored helper data to regenerate the same key. **f** Key similarity results before and after applying the fuzzy extractor.

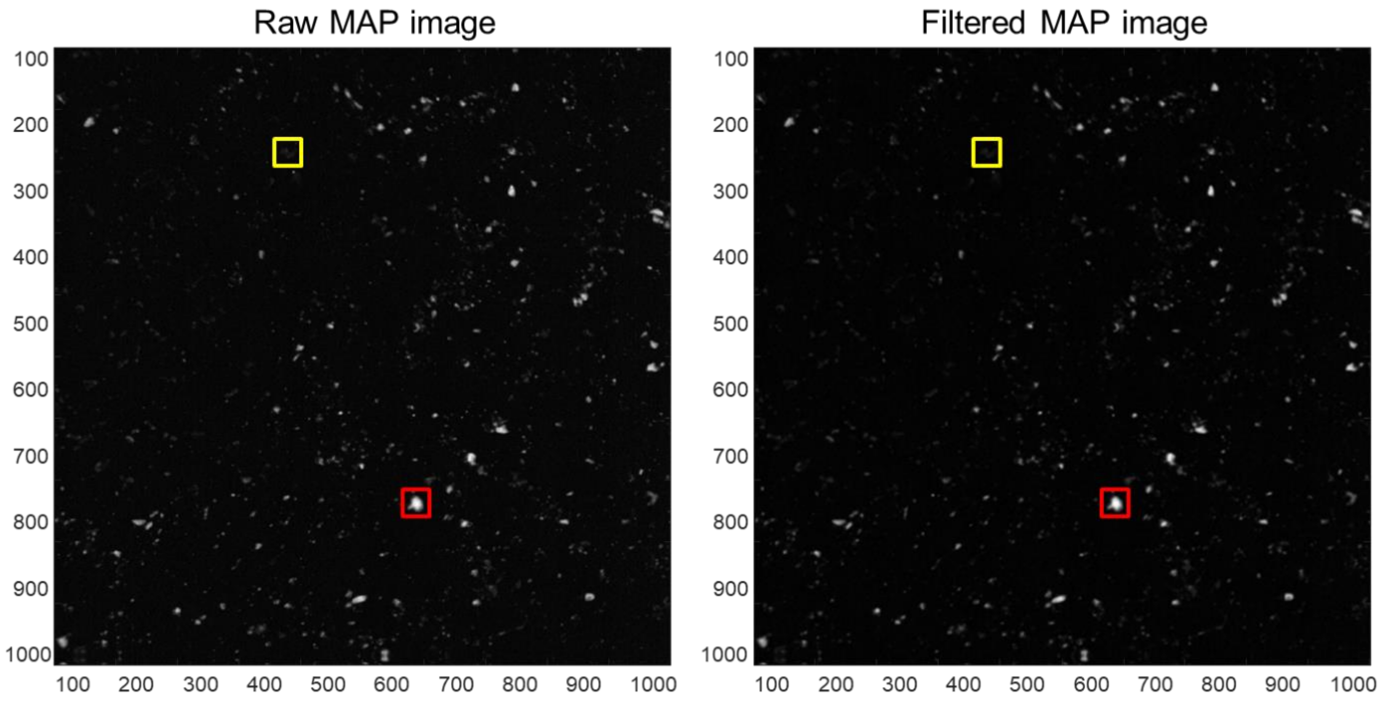

Supplementary Fig. 24 | Noise reduction in PA imaging using bandpass filtering. Comparison of raw and filtered PA images. Red and yellow ROIs indicate signal and noise regions, respectively, showing improved SNR after filtering.

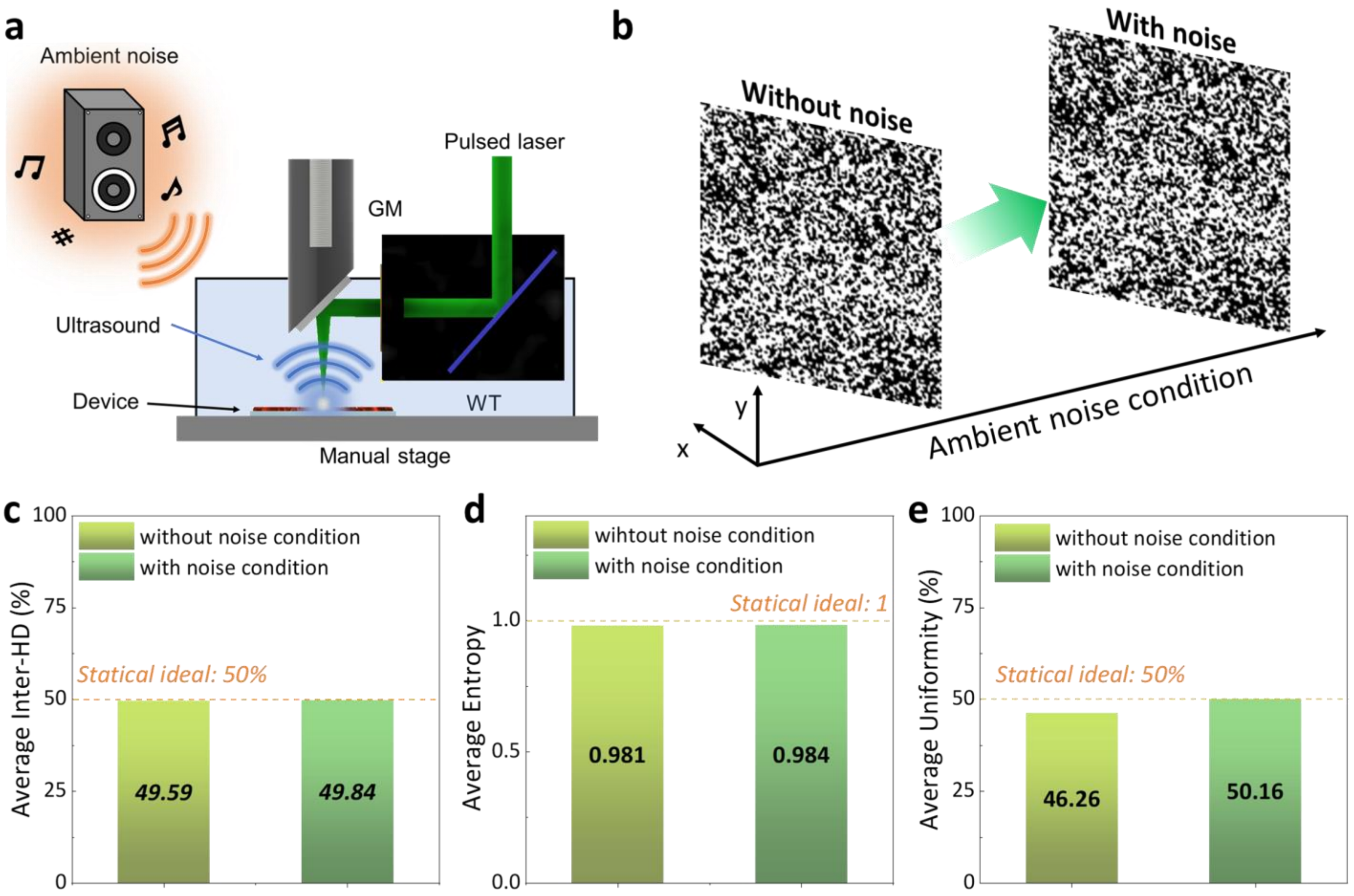

Supplementary Fig. 25 | Stability of PA-PUF responses under ambient acoustic noise. a Schematic of ambient noise test setup. **b** Digitized PA-PUF patterns acquired with and without noise exposure condition. **c** Mean inter-HD analysis results. **d** Mean entropy comparison. **e** Mean uniformity values obtained under with and without noise conditions.
