# Supplementary figures and images for: Light in, sound keys out: photoacoustic PUFs from stochastic nanocomposites
Source: Nat Commun. 2025 Aug 8;16:7323. doi: 10.1038/s41467-025-62747-1 (PMC12334560; doi:10.1038/s41467-025-62747-1)

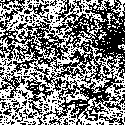

Supplement: Supplementary file 3 — Source Data [file 41467_2025_62747_MOESM3_ESM.zip › Source Data/Supplementary Figure 20/#2_30kHz_M_pixel_data_bitpattern.png]

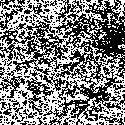

Supplement: Supplementary file 3 — Source Data [file 41467_2025_62747_MOESM3_ESM.zip › Source Data/Supplementary Figure 20/#2_40kHz_M_pixel_data_bitpattern.png]

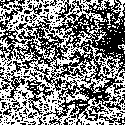

Supplement: Supplementary file 3 — Source Data [file 41467_2025_62747_MOESM3_ESM.zip › Source Data/Supplementary Figure 20/Control #2_20kHz_M_pixel_data_bitpattern.png]

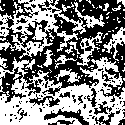

Supplement: Supplementary file 3 — Source Data [file 41467_2025_62747_MOESM3_ESM.zip › Source Data/Supplementary Figure 21/Control sample.png]

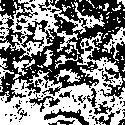

Supplement: Supplementary file 3 — Source Data [file 41467_2025_62747_MOESM3_ESM.zip › Source Data/Supplementary Figure 21/After bending.png]

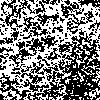

Supplement: Supplementary file 3 — Source Data [file 41467_2025_62747_MOESM3_ESM.zip › Source Data/Supplementary Figure 22/40.tif]

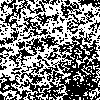

Supplement: Supplementary file 3 — Source Data [file 41467_2025_62747_MOESM3_ESM.zip › Source Data/Supplementary Figure 22/50.tif]

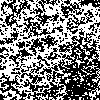

Supplement: Supplementary file 3 — Source Data [file 41467_2025_62747_MOESM3_ESM.zip › Source Data/Supplementary Figure 22/control 36.5.tif]

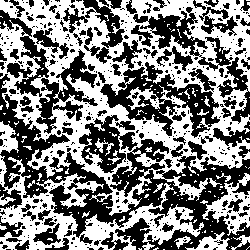

Supplement: Supplementary file 3 — Source Data [file 41467_2025_62747_MOESM3_ESM.zip › Source Data/Supplementary Figure 23/Control.png]

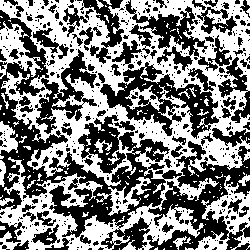

Supplement: Supplementary file 3 — Source Data [file 41467_2025_62747_MOESM3_ESM.zip › Source Data/Supplementary Figure 23/Subsequent2.png]

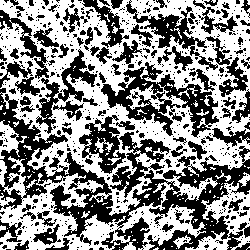

Supplement: Supplementary file 3 — Source Data [file 41467_2025_62747_MOESM3_ESM.zip › Source Data/Supplementary Figure 23/Subsequent3.png]

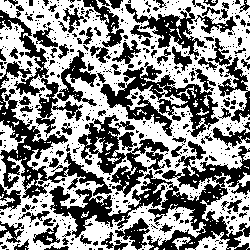

Supplement: Supplementary file 3 — Source Data [file 41467_2025_62747_MOESM3_ESM.zip › Source Data/Supplementary Figure 23/Subsequent1.png]

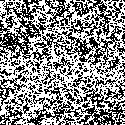

Supplement: Supplementary file 3 — Source Data [file 41467_2025_62747_MOESM3_ESM.zip › Source Data/Supplementary Figure 25/control.png]

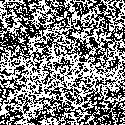

Supplement: Supplementary file 3 — Source Data [file 41467_2025_62747_MOESM3_ESM.zip › Source Data/Supplementary Figure 25/with acoustic noise.png]
